# *Eoscyphella luciurceolata* gen. and sp. nov. (Agaricomycetes) Shed Light on Cyphellopsidaceae with a New Lineage of Bioluminescent Fungi

**DOI:** 10.3390/jof9101004

**Published:** 2023-10-12

**Authors:** Alexandre G. S. Silva-Filho, Andgelo Mombert, Cristiano C. Nascimento, Bianca B. Nóbrega, Douglas M. M. Soares, Ana G. S. Martins, Adão H. R. Domingos, Isaias Santos, Olavo H. P. Della-Torre, Brian A. Perry, Dennis E. Desjardin, Cassius V. Stevani, Nelson Menolli

**Affiliations:** 1 IFungiLab, Departamento de Ciências da Natureza e Matemática (DCM), Subárea de Biologia (SAB), Instituto Federal de Educação, Ciência e Tecnologia de São Paulo (IFSP), Campus São Paulo (SPO), São Paulo 01109-010, SP, Brazil; silvafilhoags@gmail.com (A.G.S.S.-F.); cristiano.nascimento@ifpi.edu.br (C.C.N.); 2 Independent Researcher, 25640 Corcelle-Mieslot, France; mombertan@gmail.com; 3 Departamento de Bioquímica, Instituto de Química, Universidade de São Paulo, São Paulo 05508-000, SP, Brazil; bianca.barros.nobrega@usp.br; 4 Departamento de Química Fundamental, Instituto de Química, Universidade de São Paulo, São Paulo 05508-000, SP, Brazil; douglas@iq.usp.br; 5 Instituto de Pesquisa da Biodiversidade (IPBio), Iporanga 18330-000, SP, Brazil; anaglaucia@ipbio.org.br (A.G.S.M.); henrique.domingos@ipbio.org.br (A.H.R.D.); tatubio@hotmail.com (I.S.); olavopetrucci@gmail.com (O.H.P.D.-T.); 6 Department of Biological Sciences, California State University, East Bay, Hayward, CA 94542, USA; brian.perry@csueastbay.edu; 7 Department of Biology, San Francisco State University, San Francisco, CA 94132, USA; ded@sfsu.edu

**Keywords:** Agaricales, Basidiomycota, Brazilian biodiversity, bioluminescence, Niaceae

## Abstract

During nocturnal field expeditions in the Brazilian Atlantic Rainforest, an unexpected bioluminescent fungus with reduced form was found. Based on morphological data, the taxon was first identified as belonging to the cyphelloid genus *Maireina*, but in our phylogenetic analyses, *Maireina* was recovered and confirmed as a paraphyletic group related to genera *Merismodes* and *Cyphellopsis*. *Maireina filipendula*, *Ma. monacha*, and *Ma. subsphaerospora* are herein transferred to *Merismodes*. Based upon morphological and molecular characters, the bioluminescent cyphelloid taxon is described as the new genus *Eoscyphella*, characterized by a vasiform to urceolate basidiomata, subglobose to broadly ellipsoid basidiospores, being pigmented, weakly to densely encrusted external hyphae, regularly bi-spored basidia, unclamped hyphae, and an absence of both conspicuous long external hairs and hymenial cystidia. Phylogenetic analyses based on ITS rDNA and LSU rDNA support the proposal of the new genus and confirm its position in Cyphellopsidaceae. *Eoscyphella luciurceolata* represents a new lineage of bioluminescent basidiomycetes with reduced forms.

## 1. Introduction

Agaricomycetes forms a large and diverse group that includes the mushroom-forming fungi and produces the most complex basidiomata forms, such as gilled mushrooms, boletes, polypores, and puffballs [1]. Some species of gilled mushroom are well known and stand out for their natural light emission with a luciferin/luciferase chemical reaction [2,3]. The bioluminescent fungi are morphologically well characterized and typically known for their gilled or poroid basidiomata within the order Agaricales [4]. The known bioluminescent mushrooms are distributed in tropical and temperate regions, where they grow on moist decaying wood or leaves [4].

The first reports describing light emission with fungi were written in the 19th century by J. F. Heller [5]. In the 20th century, approximately 50 species of fungi related to light emission were described [6,7,8,9,10,11,12,13,14,15,16,17,18,19,20,21]. These species were evaluated and revised by Desjardin et al. [4], who recovered 64 valid names of bioluminescent mushrooms. In recent years, the number of known species has increased substantially [22,23,24,25,26,27,28,29], with approximately 110 bioluminescent fungi currently recognized [30].

Desjardin et al. [4,24] proposed four molecular lineages to accommodate the bioluminescent species: *Armillaria*, Mycenoid, *Omphalotus*, and Lucentipes. The *Armillaria* lineage is represented by species of the genus *Armillaria* (Fr.) Staude, which is phylogenetically positioned in the Physalacriaceae Corner [31]. These species are popularly called Honey Mushrooms and represent saprotrophic or forest tree root pathogens [32]. The Mycenoid is the most diverse lineage, with species known in the genus *Mycena* (Pers.) Roussel *sensu lato*, *Filoboletus* Henn. (manipularis group), *Panellus* P. Karst. (*Panellus*/*Dictyopanus* species), *Roridomyces* Rexer, and *Resinomycena* Redhead & Singer, all anchored in the family Mycenaceae Overeem [4,25]. Bioluminescent species of the Mycenoid lineage exhibit wide phenotypic variation; the majority produce small mushrooms with lamellate hymenophores, like most *Mycena* species, whilst other have poroid hymenophores like those in *Filoboletus*, and pleurotoid species with poroid or lamellate hymenophores are represented by the genus *Panellus* [4]. The *Omphalotus* lineage is well represented by species of the genera *Neonothopanus* R.H. Petersen & Krisai and *Omphalotus* Fayod plus *Nothopanus eugrammus* (Mont.) Singer [=*Pleurotus eugrammus* (Mont.) Dennis] and *Pleurotus decipiens* Corner [4]. *Omphalotus* and *Neonothopanus* are phylogenetically positioned in Omphalotaceae Bresinsky, while the phylogenetic position of *N. eugrammus* and *P. decipiens* has not yet been confirmed [4,33]. *Omphalotus* and *Neonothopanus* are saprobic, forming large and conspicuous agaricoid mushrooms, some popularly known as jack-o’-lantern mushrooms in Europe and North America [34]. *Gerronema viridilucens* Desjardin, Capelari & Stevani and *Mycena lucentipes* Desjardin, Capelari & Stevani (the Lucentipes clade) form an independent lineage of bioluminescent fungi with uncertain phylogenetic position at the family level [24,35].

Agaricomycetes also includes species that produce reduced forms such as the cyphelloid fungi, comprised primarily of saprobic species, producing minute barrel-, cup-, bowl-, or tube-shaped basidiomata with smooth and even hymenophores [36,37,38,39,40]. The cyphelloid fungi were first grouped in the artificial family Cyphellaceae Burnett [41]. The name Porotheleaceae Murrill was later related to tubular and discoid Hymenomycetes [42]. Some authors, including Cooke [43], used this classification for the reduced forms. However, the polyphyletic status of the cyphelloid fungi, with multiple lineages in the order Agaricales, has already been elucidated in previous molecular phylogenetic studies [31,44,45,46,47,48,49,50]. 

The diversity of cyphelloid fungi includes roughly 120 taxa that have been classified in approximately 40 widely accepted genera [44,51], with additional new taxa recently described [50,52,53,54,55,56]. It is estimated that the number of cyphelloid fungi distributed worldwide could reach nearly 400 to 500 species [45,53,57]. 

Currently, Cyphellopsidaceae Jülich and Niaceae Jülich are the names related to the Nia clade [45,58]. Cyphellopsidaceae is the most diverse family and the largest lineage of cyphelloid forms confirmed with molecular data [45]. The genera *Calathella* D.A.Reid. *Cyphellopsis* Donk, *Merismodes* Earle (abbreviated here as *Me*.), and *Woldmaria* W.B. Cooke were previously classified in Cyphellopsidaceae [58] and typified with *Cyphellopsis anomala* (Pers.) Donk. Niaceae was erected in the same work [58] to accommodate the genus *Nia* R.T. Moore & Meyers, typified by the marine species *Nia vibrissa* R.T. Moore & Meyers. Binder et al. [59] placed *N. vibrissa* in the euagaric clade and Hibbett and Binder [60] confirmed its placement in the euagaric clade along with two additional marine basidiomycetes, *Calathella mangrovei* E.B.G. Jones & Agerer and *Halocyphina villosa* Kohlm. & E. Kohlm. Bodensteiner et al. [45] recognized in the Nia clade the cyphelloid genera *Calathella*, *Cyphellopsis*, *Flagelloscypha* Donk, *Halocyphina* Kohlm. & E. Kohlm., *Lachnella* Fr., *Merismodes*, and *Woldmaria*, as well as the corticioid genus *Dendrothele* Höhn. & Litschn. Finally, *Maireina* W.B. Cooke (abbreviated here as *Ma*.) has had its phylogenetic position confirmed in the Nia clade (=Cyphellopsidaceae) [54,55]. In the Mycobank and the Index Fungorum databases, the names *Digitatispora* Doguet, *Flagelloscypha*, *Halocyphina*, *Lachnella*, *Maireina*, *Merismodes*, *Nia*, *Peyronelina* P.J. Fisher, J. Webster & D.F. Kane, and *Woldmaria* are still classified in the family Niaceae. The name Cyphellopsidaceae was legitimized over Niaceae by Knudsen and Vesterholt [61], although the name Niaceae is still being used by some authors (e.g., [62]).

During one of many nocturnal expeditions into the Atlantic Rainforest in the state of São Paulo (Brazil), in the same area where 12 bioluminescent species have already been described or recorded [22,23,24,25], an unusual bioluminescent fungus with cyphelloid form was discovered by co-authors of this work. The aims of this study are as follows: (i) confirm the phylogenetic position and classification of all known bioluminescent fungi based on molecular data; (ii) identify, based on morphology and molecular data, the new bioluminescent fungi with reduced form; and (iii) provide the phylogenetic placement of *Maireina monacha* to better understand its relationship with related genera. Based on molecular analyses, *Maireina* is considered a synonym of *Merismodes* and is herein amended. *Maireina filipendula* Læssøe, *Ma. monacha* (Speg.) W.B. Cooke, and *Ma. subsphaerospora* Mombert are transferred to *Merismodes,* and the new bioluminescent cyphelloid taxon from Brazil is described in the new genus *Eoscyphella* gen. nov., within Cyphellopsidaceae. *Eoscyphella luciurceolata* represents a new lineage of bioluminescent basidiomycetes with cyphelloid form.

## 2. Materials and Methods

### 2.1. Collecting Area

#### 2.1.1. Brazilian Site of the New Luminescent Taxon

Basidiomata of the new bioluminescent taxon were collected during expeditions to the Atlantic Rainforest in the municipality of Eldorado, state of São Paulo, Brazil. More specifically, at a 546 m altitude and 500 m west of the entrance to the “Caverna do Diabo” (Devil’s Cave) State Park at coordinates 24°38′14.0100” S and 48°24′37.6812” W. The climate there is classified as humid subtropical, and the mean annual temperatures are usually between 20 and 22 °C and have a high pluviometric index, with average annual rainfall ranging from 1500 to 2000 mm [63]. The forest type is Dense Ombrophilous Forest, which is mainly composed of the Angiosperm families Annonaceae Juss., Euphorbiaceae Juss., Lauraceae Juss., Melastomataceae Juss., Moraceae Gaudich., Myrtaceae Juss., Rubiaceae Juss., and Sapotaceae Juss. [64,65].

#### 2.1.2. French Site of *Maireina monacha*

The Butte de la Garenne is located in the Cantal department in Southern-Central France. The site is covered with a calcareous beech forest of approximately one hectare, and a pubescent oak forest in the remaining area [66]. 

### 2.2. Morphological Analyses

Macroscopic features were recorded from fresh material. Color names and codes follow Kornerup and Wanscher [67]. Micromorphological analyses were performed using the methodology of Bodensteiner [53]. Basidiospores were measured in lateral view using 5% KOH. Basidiospore statistics include the following: xm = arithmetic mean of basidiospore length × basidiospore width (±standard deviation) for *n* basidiospores measured in a single specimen; xr = range of basidiospore means; Q = quotient of basidiospore length by basidiospore width in any one basidiospore, indicated as a range of variation in *n* basidiospores measured; Qm = mean of Q-values in a single specimen; *n* = number of basidiospores measured per specimen; and s = number of specimens studied. Distilled water was used in order to visualize crystals in skeletal hyphae, whilst Melzer’s reagent was used to test amyloid/dextrinoid reactions. The Brazilian specimens were deposited at the Fungarium IFungi (FIFUNGI) from the IFungiLab at the “Instituto Federal de Educação, Ciência e Tecnologia de São Paulo (IFSP)”, Brazil, and the European specimens are housed at the “Muséum National d’Histoire Naturelle” (P), France ([68], 2023, continuously updated).

### 2.3. Molecular Methods

Entire basidiomata were homogenized in lysis tubes with magnetic beads for three cycles of 2 min in SpeedMill Plus (Analytik, Jena, Germany) in an AP1 buffer, and the genomic DNA was extracted using the Qiagen Dneasy^®^ Plant Mini Kit (Germantown, MD, USA) according to the manufacturer’s instructions. Primer pairs ITS1-F/ITS4 and LR0R/LR5 were used to amplify and sequence the ITS rDNA region and the LSU rDNA gene, respectively [44,69]. Sequencing reactions were conducted at Macrogen (Seoul, Republic of Korea).

### 2.4. Phylogenetic Analyses

The newly generated sequences were assembled and edited in Sequencher TM v5.0 software (Gene Codes Corporation, Ann Arbor, MI, USA) and were deposited in GenBank (codes in the tree and in Supplementary Table S1). Three new ITS rDNA and two novel LSU r DNA sequences were generated in this study. Three distinct datasets were constructed: one composed only of the LSU rDNA sequences, one only with ITS rDNA sequences, and a third including the ITS rDNA + LSU rDNA sequences. To assemble the LSU rDNA dataset, our generated sequences were submitted to the BLASTn algorithm at NCBI (GenBank, https://blast.ncbi.nlm.nih.gov/Blast.cgi, accessed on 1 May 2023) to retrieve similar sequences. Other sequences of cyphelloid basidiomycetes, including those generated by Bodensteiner et al. [45], Læssøe et al. [54], Baltazar et al. [48], Karasiński et al. [56], and Vizzini et al. [70], were downloaded and included in the dataset. Existing sequences of known bioluminescent fungi were also downloaded from GenBank to compose a final dataset that includes all known bioluminescent and cyphelloid lineages. The LSU rDNA sequence most likely misnamed *G. viridilucens* (EF514207), which is available on GenBank and was used in the phylogenetic analyses by Vizzini et al. [70] and Na et al. [71], is 99.7% identical to sequence *M. lucentipes* (DED7828) [72]. For this reason, we excluded the sequence EF514207 from our phylogenetic analyses and included a new one of *G. viridilucens* (DED7822), originally from type locality and morphologically described and confirmed [22]. For the ITS rDNA dataset, sequences of species belonging to Cyphellopsidaceae were retrieved from GenBank and then used to recover similar sequences using the BLASTn algorithm. The combined ITS+LSU rDNA dataset was constructed to focus primarily on the *Merismodes* clade. *Boletus griseiceps* B. Feng, Y.Y. Cui, J.P. Xu & Zhu L. Yang; *Boletus subviolaceofuscus* B. Feng, Y.Y. Cui, J.P. Xu & Zhu L. Yang; and *Fistulinella ruschii* Magnago were used as an outgroup in the LSU rDNA dataset. Two sequences of *Cunninghammyces* Stalpers were used as an outgroup in the ITS rDNA dataset, and sequences of *Acanthocorticium brueggemannii* Baltazar, Gorjón & Rajchenb were used for the combined analyses.

Our datasets were aligned using MAFFT v.7 under the E-INS-i criteria [73]. Seaview v.4 was used to visualize the alignment [74]. To compute the best-fit model of nucleotide evolution, the ITS rDNA alignment was subdivided into three partitions: ITS1, 5.8S, and ITS2. Maximum Likelihood analyses were performed in RAxML v8.2.X [75]. The most appropriate nucleotide substitution models were selected with BIC (Bayesian Information Criterion) using jModelTest 2v.1.6 [76]. Bayesian inferences (BI) were performed using MrBayes 3.1.2, performing 2 × 10^7^ MCMC generations, sampling one tree every 1 × 10^3^ generations [77]. The jModelTest 2v.1.6, RAxML v8.2.X, and MrBayes 3.1.2 software were implemented in CIPRES Science Gateway 3.1 [78]. Trees were visualized and rooted in FigTree v.1.4.4 and the final tree figures were completed in CorelDRAW Grafics Suit 2021. A node was considered significantly supported if it received bootstrap (BS) ≥ 70% and Bayesian posterior probability (BPP) ≥ 0.95.

## 3. Results

### 3.1. Phylogenetic Results

#### 3.1.1. LSU rDNA Dataset

**Figure 1:**
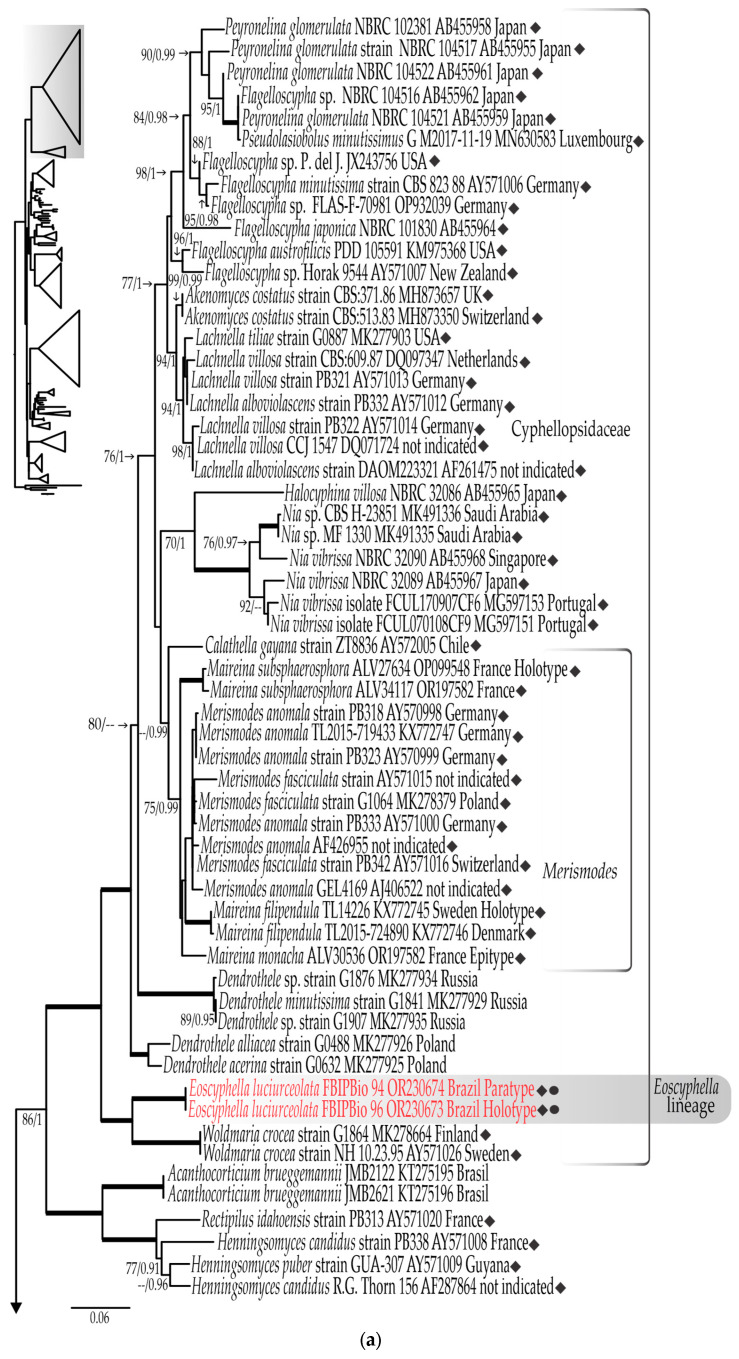

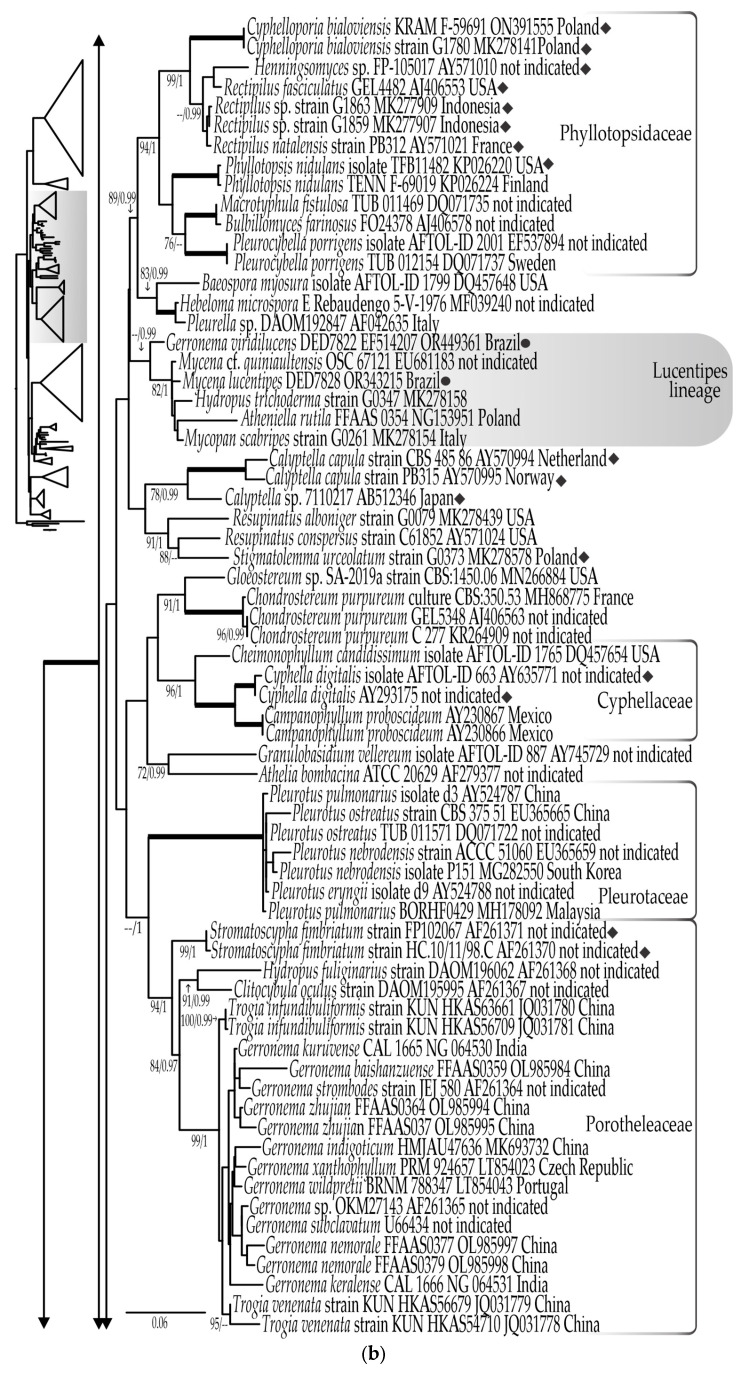

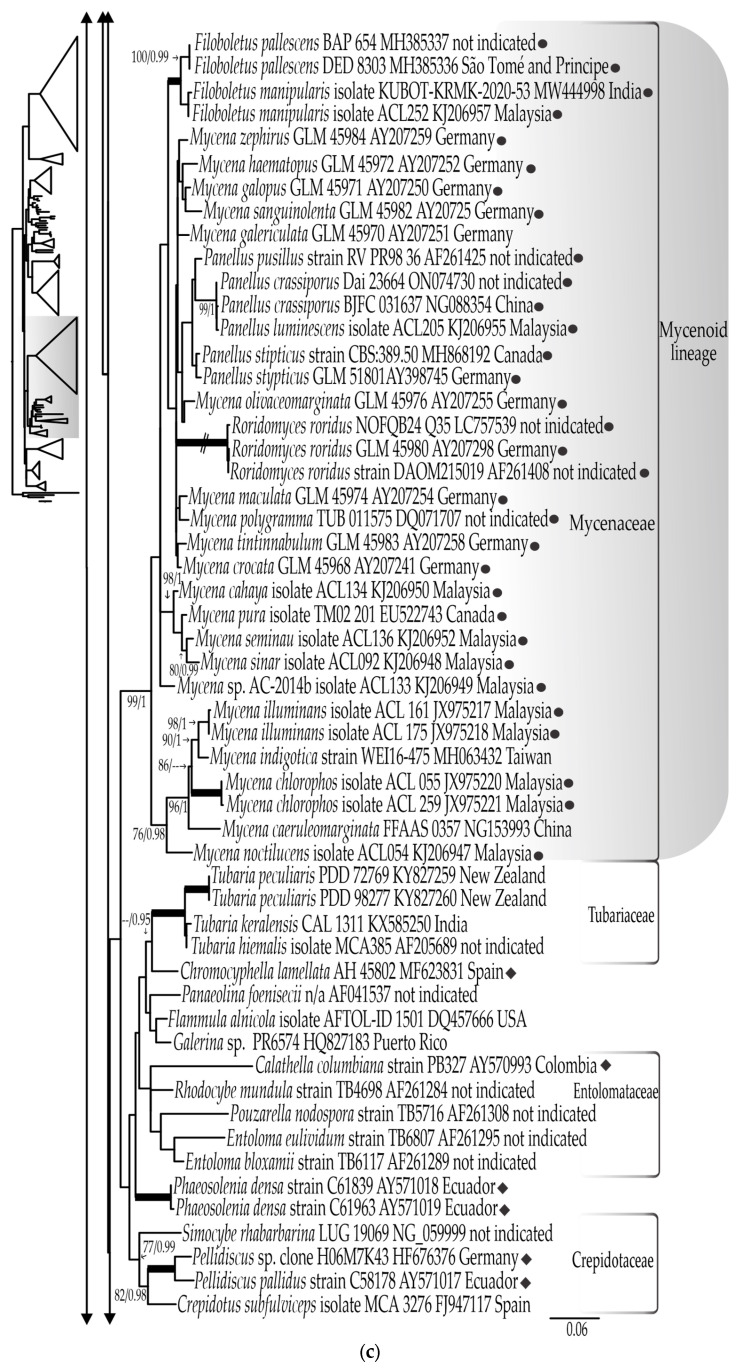

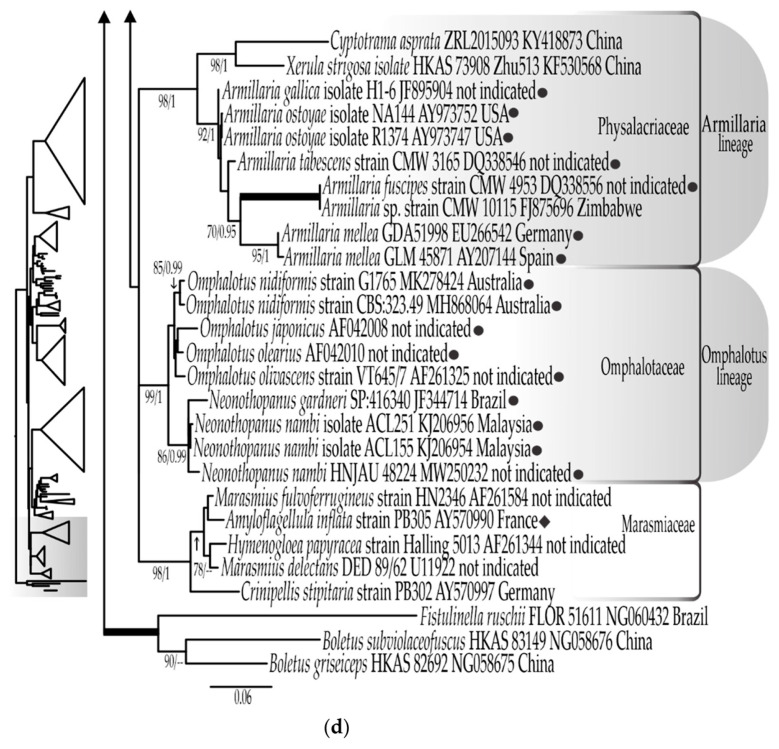
(**a**–**d**) The ML phylogeny of representative collections of Agaricomycetes based on complete LSU rDNA. Voucher/strain/clone or herbarium number as well as GenBank accession numbers and country of origin follow taxon name. Cyphelloid species are noted with the symbol ◆ and bioluminescent species with the symbol ⚫. The new species is highlighted in red, and the luminescent lineages are in gray. Thicker lines represent branches with maximum bootstrap values and posterior probabilities (100% BS, 1.0 BPP). Bootstrap values and Bayesian posterior probabilities are indicated if they are equal to or greater than 70%, and 0.95, respectively. The scale bar represents the expected number of nucleotide changes per site.

The final LSU rDNA dataset contains 206 sequences (including 2 that are newly generated), consisting of 1051 nucleotide sites, including gaps. The most appropriate evolutionary model estimated was TrN+I+G. The bootstrapping criteria from the ML analyses stopped after 350 replicates. Both the RAxML analysis and Bayesian inference yielded similar tree topologies. The LSU rDNA tree generated from the ML analysis, including bootstrap values and posterior probabilities, is shown in four parts (Figure 1a–d).

The family Cyphellopsidaceae, represented by 52 sequences, forms a well-supported clade (100% BS, 1.0 BPP) (Figure 1a) that harbors the largest number of cyphelloid genera hitherto confirmed with molecular data: *Akenomyces* G. Arnaud, *Calathella* (represented by *Calathella gayana* (Lév.) Agerer), *Flagelloscypha*, *Halocyphina*, *Lachnella*, *Maireina*, *Merismodes*, *Nia*, *Eoscyphella* gen. nov., *Pseudolasiobolus* Agerer, and *Woldmaria*.

The new proposed genus *Eoscyphella* formed a well-supported clade (100% BS, 1.0 BPP) sister to the cyphelloid genus *Woldmaria*. The LSU rDNA sequences of *Eoscyphella* and *Woldmaria* are from 92.6% to 92.7% similar. Sequences of taxa of the genus *Maireina* were represented in our analyses by *Ma. filipendula, Ma. subsphaerosphora*, and *Ma. monacha* (type species of *Maireina*), with the latter sampled, sequenced, and identified by Mombert [55] and fully described and epityfied in this study. *Maireina* formed a paraphyletic group, but represents a monophyletic clade when including sequences of *Merismodes anomala* (Pers.) Singer (as = *Cyphellopsis anomala*) and *Me. fasciculata* (Schwein.) Earle, with the latter being the type species of the genus. The clade formed with *Maireina* and *Merismodes* is well supported (75% BS, 0.99 BPP).

In addition to Cyphellopsidaceae, our LSU rDNA analyses recovered another 11 lineages of cyphelloid fungi (Figure 1a–d). Several cyphelloid genera are recovered in distinct well-supported clades that correspond to at least five well-delimited families: in Cyphellaceae (96% BS, 1.0 BPP), the genus *Cyphella* Fr. (100% BS, 1.0 BPP); in Crepidotaceae (S. Imai) Singer (77% BS, 0.99 BPP), the genus *Pellidiscus* Donk (100% BS, 1.0 BPP); in Marasmiaceae Roze ex Kühner (98% BS, 1.0 BPP), the genus *Amyloflagellula* Singer; in Phyllotopsidaceae Locquin ex Olariaga, Huhtinen, Læssøe, J.H. Petersen & K. Hansen (94% BS, 1.0 BPP), the genus *Cyphelloporia* Karasiński, L. Nagy, Szarkándi, Holec & Kolařík (100% BS, 1.0 BPP); and in Porotheleaceae (94% BS, 0.99 BPP), the genus *Stromatoscypha* Donk (100% BS, 1.0 BPP). The cyphelloid genus *Stigmatolemma* Kalchbr. clusters with sequences of *Resupinatus alboniger* (Pat.) Singer (MK278432) and *Resupinatus conspersus* (Pers.) Thorn, Moncalvo & Redhead (AY570994) in a well-supported clade (91% BS, 1.0 BPP) that is sister (unsupported) to the well-supported clade (78% BS, 0.99 BP) formed with sequences of the cyphelloid genus *Calyptella* Quél. (Figure 1b).

Additionally, sequences of the cyphelloid genera *Henningsomyces* Kuntze and *Rectipilus* Agerer are resolved in two phylogenetically distant clades: the *Henningsomyces*/*Rectipilus*/*Acanthocorticium* clade (100% BS, 1.0 BPP) that is sister (86% BS, 1.0 BPP) to Cyphellopsidaceae (Figure 1a), and in the clade of Phyllotopsidaceae *sensu* Olariaga et al. [77] and Karasiński et al. [56], forming a well-supported clade (99% BS, 1.0 BPP) with *Cyphelloporia* representatives (Figure 1b). Other cyphelloid taxa, such as *Calathella columbiana* Agerer (AY570993), *Chromocyphella lamellata* G. Moreno & Olariaga (MF623831), and *Phaeosolenia densa* (Berk.) W.B. Cooke (AY571018, AY571019) formed independent lineages with no clear relationship to other known lineages (Figure 1c). 

The four bioluminescent lineages *sensu* Desjardin et al. [4] are represented (Figure 1a–d). The Mycenoid lineage is the largest and forms a monophyletic group in a well-supported (99% BS, 1.0 BPP) clade (family Mycenaceae) represented in our analyses (Figure 1c) by 25 species of the genera *Filoboletus*, *Mycena*, *Panellus*, and *Roridomyces*. The *Armillaria* lineage (Figure 1d) is here represented by five species of the genus *Armillaria* that clustered in a well-supported (92% BS, 1.0 BPP) clade sister to sequences of *Cyptotrama asprata* (Berk.) Redhead & Ginns (KY418873) and *Xerula strigosa* Zhu L. Yang, L. Wang & G.M. Muell. (KF305680) within Physalacriaceae (97% BS, 1.0 BPP). The *Omphalotus* lineage is represented (Figure 1d) by six species of *Omphalotus* and *Neonothopanus* that form a well-supported (98% BS, 1.0 BPP) clade corresponding to Omphalotaceae. The Lucentipes clade forms a well-supported (0.99 BPP) independent lineage (Figure 1b) that contains, in addition to *Gerronema viridilucens* (EF514207) and *Mycena lucentipes* (OR343215), sequences identified as *Atheniela rutila* Q. Na & Y.P. Ge, (NG153951), *Mycopan scabripes* (Murrill) Redhead, Moncalvo & Vilgalys (MK278154), *Hydropus trichoderma* (Joss.) Singer (MK278158), and *Mycena* cf. *quiniaultensis* Kauffman (EU681183). A fifth bioluminescent lineage is composed of the proposed new genus *Eoscyphella,* represented by two sequences of *Eoscyphella luciurceolata* sp. nov. (Figure 1a).

#### 3.1.2. ITS rDNA Dataset

**Figure 2:**
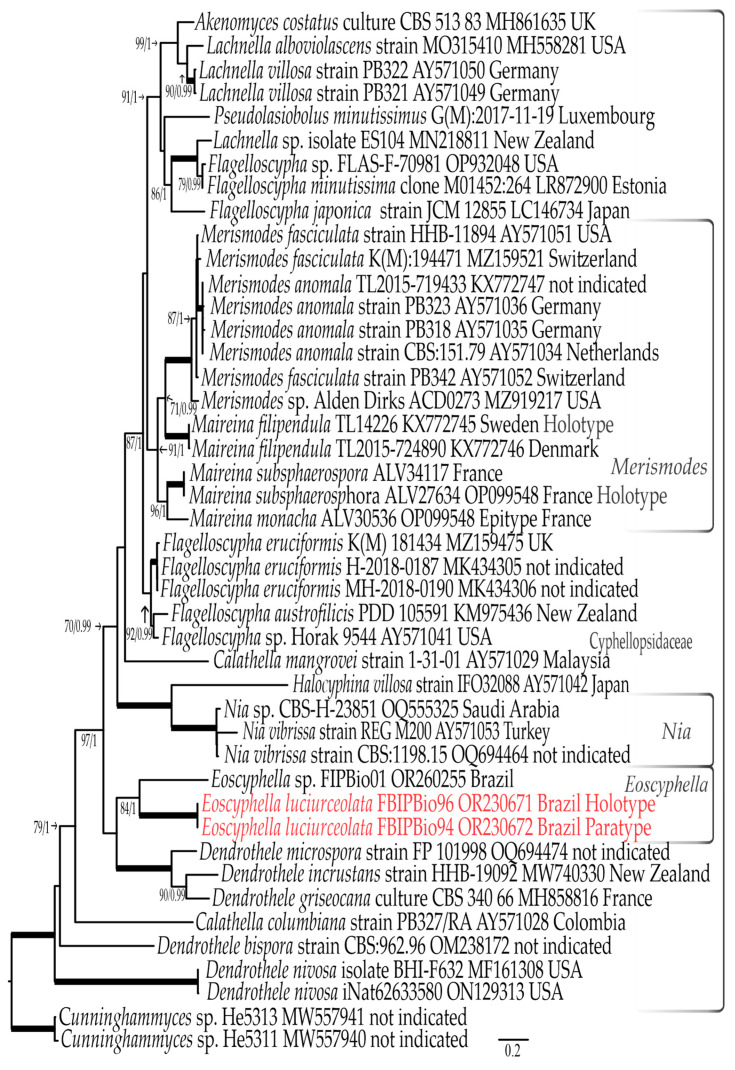
ML phylogeny of collections of Cyphellopsidaceae representatives based on complete ITS rDNA. The new species is highlighted in red. Voucher/strain/clone or herbarium number as well as GenBank accession numbers and country of origin follow taxon name. Thicker lines represent branches with maximum bootstrap values and posterior probabilities (100% BS, 1.0 BPP). Bootstrap values and Bayesian posterior probabilities are indicated if they are equal to or greater than 70%, and 0.95, respectively. The scale bar represents the expected number of nucleotide changes per site.

The final ITS rDNA dataset has 44 sequences (including 3 that are newly generated), consisting of 1051 nucleotide sites, including gaps. The best evolutionary models estimated for each part of the alignments were ITS1: TPM2uf+G, 5.8S: TPM2+G, and ITS2: HKY+G. The bootstrapping criteria from the ML analysis stopped after 300 replicates. Both the RAxML analysis and Bayesian inference yielded similar tree topologies. The ITS rDNA tree generated from the ML analysis, including bootstrap and posterior probabilities, is shown in Figure 2.

The family Cyphellopsidaceae (100% BS, 1.0 BPP) is represented by 42 sequences, with no representatives of *Woldmaria* nor *Peyronelina* due to lack of available sequences. *Eoscyphella luciurceolata* sp. nov. and the non-bioluminescent *Eoscyphella* sp. formed a well-supported clade (84% BS, 1.0 BPP), sister to (but not supported) a clade that contains sequences of *Dendrothele microspora* (H.S. Jacks. & P.A. Lemke) P.A. Lemke, *Dendrothele incrustans* (P.A. Lemke) P.A. Lemke, and *Dendrothele griseocana* (Bres.) Bourdot & Galzin (Figure 2). The genus *Maireina*, represented by the same species as in the LSU rDNA analyses, is again confirmed as paraphyletic with the ITS rDNA data. However, as in the nLSU analyses, the included *Maireina* sequences form a monophyletic and well-supported clade (91% BS, 1.9 BPP) when including sequences of *Merismodes anomala*, *Me. fasciculata,* and *Merismodes* sp. (MZ919217).

#### 3.1.3. Combined LSU rDNA + ITS rDNA Dataset

The final combined LSU rDNA plus ITS rDNA dataset contains 20 ITS rDNA and 16 LSU rDNA sequences (including 5 generated as part of this study) for 21 terminals, and consists of 1806 nucleotide sites, including gaps. The most appropriate evolutionary models estimated for each part of the alignments were ITS1: TPM2uf+G, 5.8S: TPM2, ITS2: TPM2uf+G, and LSU: TIM3+G.

**Figure 3:**
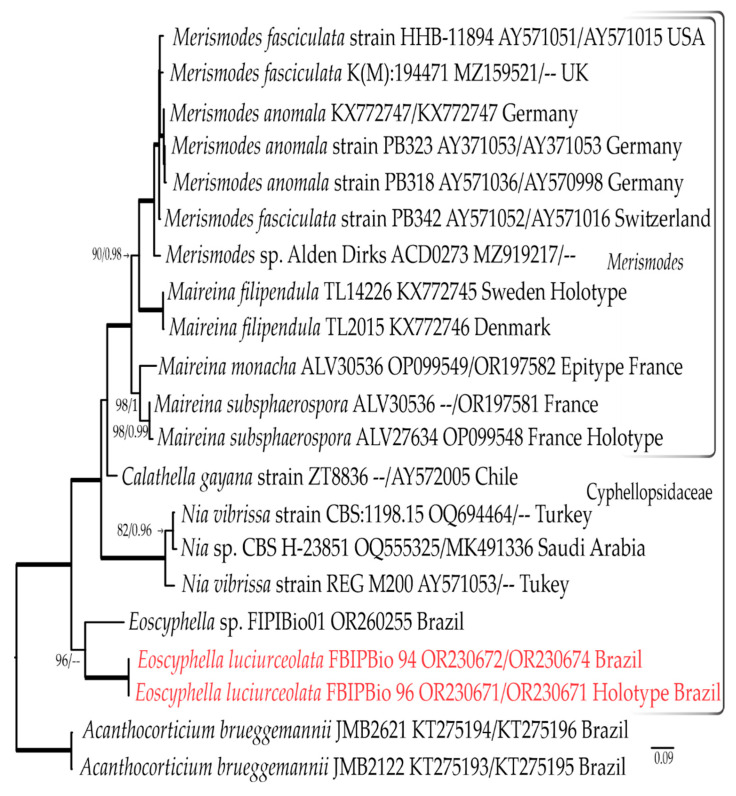
ML phylogeny of Cyphellopsidaceae focusing on collections of *Merismodes* representatives based on combined ITS rDNA and LSU rDNA. The new species is highlighted in red. Voucher/strain/clone or herbarium number as well as GenBank accession numbers and country of origin follow taxon name. Thicker lines represent branches with maximum bootstrap values and posterior probabilities (100% BS, 1.0 BPP). Bootstrap values and Bayesian posterior probabilities are indicated if they are equal to or greater than 70%, and 0.95, respectively. The scale bar represents the expected number of nucleotide changes per site.

The bootstrapping criteria from the ML analysis stopped after 50 replicates. The most likely tree generated with the ML analysis is shown in Figure 3. The family Cyphellopsidaceae (100% BS, 1.0 BPP) is represented by 19 terminals, with emphasis on the *Merismodes* clade (100% BS, 1.0 BBP), represented by 12 terminals of *Maireina* and *Merismodes*. Consistent with the other previous analyses, both the Bayesian inference and ML analysis recover *Maireina* as a paraphyletic group (Figure 3).

### 3.2. Taxonomic Part

From molecular phylogenetic results, we consider *Maireina* a synonym of *Merismodes* (=*Cyphellopsis*), supporting the taxonomic concept of Knudsen and Vesterholt [61], who considered *Maireina, Cyphellopsis*, and *Phaeocyphellopsis* W.B. Cooke synonyms of *Merismodes*. We herein propose the combination of *Ma. monacha*, *Ma. filipendula*, and *Ma. subsphaerophora* into *Merismodes*, as well as the description of the genus *Eoscyphella* to accommodate the novel bioluminescent cyphelloid species from Brazil.

***Merismodes*** Earle, Bulletin of the New York Botanical Garden 5: 406 (1909) emend. Silva-Filho & Menolli

=*Cyphellopsis* Donk, Mededelingen van de Nederlandse Mycologische Vereeniging 18–20: 128 (1931).

=*Maireina* W.B. Cooke, Beihefte zur Sydowia 4: 83 (1961).

*=Phaeocyphellopsis* W.B. Cooke, Beihefte zur Sydowia 4: 119 (1961). 

=*Pseudodasyscypha* Velen., Novitates mycologicae: 167 (1939).

*Original diagnosis* [79]: Not pultrecent, densely connate-cespitose: pileus fleshy, irregular: lamellae reduced to obscure folds: spores white or hyaline: veil none: stipe irregular, the bases fused.

Emended description: Basidiomata gregarious or scattered. Receptacle cyphelloid, cupulate to tubular, sessile or pendant; outside covered with yellow brown to brown hairs, hymenium pale, whitish. Subiculum absent or developed. External hyphae thick-walled, not branched, straight, attenuated to spiraled towards the distal end, yellow to brown pigmented, sometimes with apical ends colorless, tips incrusted or smooth, obtuse to inflated, inamyloid to slightly dextrinoid. Trama gelatinous or non-gelatinous. Basidiospores subglobose, ellipsoid, cylindrical, allantoid or subfusiform, smooth, thin-walled, hyaline, inamyloid. Basidia cylindrical to clavate, four-spored, occasionally two-spored. Cystidia absent or rarely present. Clamp connections present or absent.

Notes: After the very brief protologue, Knudsen and Vesterholt [61] included in their description of *Merismodes* include some morphological characteristics of the genera *Maireina*, *Cyphellopsis*, and *Phaeocyphellopsis*. In our emendation, we include additional distinctive morphological characteristics of the species recently described [52,53,54] and of *Maireina* based on Bodensteiner [57]. In all our analyses, the genus *Maireina* is resolved as paraphyletic, forming a well-supported monophyletic lineage with the sequences of *Mersimodes* included. Based on these results and those of previous investigators [61], we consider *Maireina* a synonym of the latter genus and propose an amendment. The name *Merismodes*, proposed in 1909 [79], has priority against *Maireina* erected in 1961 [43]. Thus, to better accommodate the *Maireina* species sampled in our analyses (which includes sequences from holotype material), we propose the combination of *Ma. filipendula* and *Ma. subsphaerosphora* in *Merismodes*. Additionally, a recently collected sample of *Ma. monacha* (type species of *Maireina*) from France (same country locality of the holotype) was also included in our analyses. The taxon is herein re-analyzed and confirmed in *Merismodes* and an epitype is designated.

***Merismodes monacha*** (Speg.) Silva-Filho, Mombert & Menolli comb. nov.
MycoBank: MB 849402

Figure 4a–d

*Basionym*: *Cyphella monacha* Speg., Michelia 2 (7): 303 (1881).

≡*Cyphellopsis monacha* (Speg.) D.A. Reid, Kew Bulletin 17: 297 (1963).

≡*Maireina monacha* (Speg.) W.B. Cooke, Beihefte zur Sydowia 4: 90 (1961).

*=Cyphella bresadolae* Grélet, Bulletin de la Société Mycologique de France 38: 174 (1922). 

*=Cyphella bresadolae* var. *gregaria* (Syd. & P. Syd.) Pilát, Annales Mycologici 23: 162 (1925). 

*=Cyphella bresadolae* var. *leochroma* (Bres.) Grélet, Bulletin de la Société Mycologique de France 38: 174 (1922).

*=Cyphella bresadolae* var. *tephroleuca* (Bres.) Grélet, Bulletin de la Société Mycologique de France 38: 174 (1922). 

*=Merismodes bresadolae* (Grélet) Singer, The Agaricales in modern taxonomy. 3rd ed. J. Cramer, Lehre, Vaduz: 665 (1975). 

*=Cyphella gregaria* Syd. & P. Syd., Hedwigia 39(3): 116 (1900). 

*=Cyphella leochroma* Bres., Fungi Tridentini II (fasc. 14): 99, Table 211, f. 1 (1900).

*=Cyphella obscura* Roum., Fungi selecti gallici exsiccati. Michelia II, Cent. 20, no. 1905 (1882). 

*=Cyphella sydowii* Bres., in SYDOW H, Mycotheca Marchica. Cent. 38, no. 3706 (1892). 

*=Cyphella tephroleuca* Bres., Fungi Tridentini II (fasc. 11–13): 57, Table 166, f. 2 (1898). 

*=Maireina marginata* (McAlpine) W.B. Cooke, Sydowia, Annales Mycologici, Beiheft 4: 89 (1962).

Macro- and micro-morphological description: Cooke [43].

Material examined: FRANCE, Cantal. St-Santin-de-Maurs, on a still-attached dead twig of *Cornus sanguinea* L., 28 June 2021. Leg. A. Mombert., ALV30536 [PC0142589, Epitype here designated! (validated identifier: MBT 204394)].

Habitat and known distribution: On bark of dead branch of *Cornus sanguinea* in oak forest in France, but also *Acer campestre* L. (Aceraceae), *Berberis vulgaris* L. (Berberidaceae), *Bupleurum fruticosum* L. (Apiacaceae), *Hieracium umbellatum* L. (Asteraceae), *Cytisus* sp., *Genista tinctoria* L., *Sarothamnus scoparius* (L.) Link (Fabaceae), *Lonicera* sp. (Caprifoliaceae), *Prunus amygdalus* Batsch, *P. persica* (L.) Batsch (Rosaceae), *Quercus mongolica* Fisch. ex Ledeb. (Fagaceae) [57]. Distributed in Europe and Oceania [43].

Notes: Our specimen agrees with the description of *Me*. *monacha* presented by Cooke (ref. [43], as *Ma. monacha*), who analyzed authentic material of all names included here as synonyms, including the types of *Cyphella obscura* Roum. and *Cyphella sydowii* Bres. According to Cooke [43], *Me. monacha* is characterized by brown receptacles with long hairs around the cup edge and at the hymenial surface, elongate to cylindrical basidiospores, four-spored basidia, and cylindrical, yellowish brown to brown external hyphae with paler apices. Although our material has had slightly broader receptacles (1.5–3 mm diam.) and basidia (9.0–110 µm diam.) than reported by Cooke [43] (receptacles, 0.5–1 mm diam.; basidia, 5.5–8.0 µm diam.), other macro- and micromorphological characteristics are sufficient for the identification of this sample as *Ma. monacha sensu* Cooke [43] and Bodensteiner [57]. *Merismodes monacha* was originally described from samples collected in France but it has a distribution recorded in many European countries, including Germany, Austria, Italy, the Czech Republic, Hungary, the United Kingdom, and one record from Australia [43]. The holotype of *Cyphella monacha* Speg. [anon. s.n. (Fung. Gall. 768) Spegazzini s.n.] was deposited at the New York Botanical Garden Herbarium (NY). Considering the complete morphological and molecular data recovered from our sample that is from a region close to the type locality, we decided to designate the voucher ALV30536 as epitypus.

***Merismodes filipendula*** (Læssøe) Silva-Filho & Menolli comb. nov.

MycoBank: MB 849405

Basionym: *Maireina filipendula* Læssøe, Karstenia 56 (1): 40 (2016).

Macro- and micro-morphological description: see Læssøe et al. [54].

***Merismodes subsphaerospora*** (Mombert) Silva-Filho, Mombert & Menolli comb. nov.

MycoBank: MB 849406

Basionym: *Maireina subsphaerospora* Mombert, Bulletin Mycologique et Botanique Dauphiné-Savoie 246: 38 (2022).

Macro- and micro-morphological description: see Mombert [55].

***Eoscyphella*** Silva-Filho, Stevani & Menolli gen. nov.

MycoBank: MB 849403

Etymology: *Eos* = light of day; the goddess of dawn (Greek); *cyphella* (from kyfos in Greek) = shape of a cup, something hollow. The prefix “Eos” is in reference to the light emitted by the bioluminescent basidiomata of the type species. Additionally, the Roman equivalent refers to *Eosforos* as Lucifer, which is the entity’s name that was later considered into Christianity as the devil, and it also refers to the name of the protected area (Devil’s Cave State Park) near where the specimens of the type species were found. The name cyphella is a reference to the genus *Cyphella* and to the cyphelloid body form. 

Type species: *Eoscyphella luciurceolata* Silva-Filho, Stevani & Desjardin (described below).

Diagnosis: *Eoscyphella* is morphologically similar to *Merismodes* and *Woldmaria* but differs from *Woldmaria* in lacking conspicuous long hairs in the receptacle, subglobose to broadly ellipsoid basidiospores, regularly bi-spored basidia, and unclamped hyphae; and from *Merismodes* by the absence of conspicuous hairs in the receptacle, absence of cystidia, regularly bi-spored basidia, and the characteristic external hyphae that are always pigmented and encrusted at the tips. 

Notes: *Eoscyphella,* typified here using *Eoscyphella luciurceolata* sp. nov., represents a new lineage of bioluminescent fungi. It is supported with phylogenetic data (Figure 1a, Figure 2 and Figure 3) and morphological characteristics, including the absence of conspicuous long hairs on the receptacle, subglobose to broadly ellipsoid basidiospores, regularly bi-spored basidia, the absence of clamp connections, and the consistent presence of pigmented and encrusted external hyphae. An additional collection (FIPBIO 01) of a related non-bioluminescent cyphelloid species was found in the same region of the type species. The ITS rDNA sequence data (OR260255) resolves this taxon as sister to *E. luciurceolata* and suggests that it represents an additional species of *Eoscyphella* (Figure 2 and Figure 3). The presence of a second species indicates that *Eoscyphella* is likely a non-monospecific genus that includes both bioluminescent and non-bioluminescent members. Until additional material of the non-bioluminescent taxon can be collected to confirm these initial observations, we prefer to leave it undescribed.

**Figure 5:**
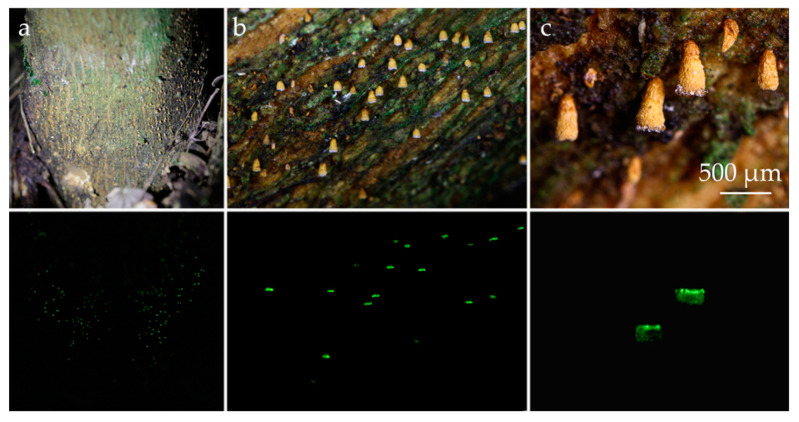
*Eoscyphella luciurceolata* basidiomata in light (above) and dark (below). (**a**–**c**) On the bark of “fumeiro” tree (*Solanum swartzianum*). Note that only dry mushrooms, whose margin is adorned with water droplets, emit light. FBIPBio 93.20220802 (Paratype–FIFUNGI00249). Photos by Adão Henrique Rosa Domingos.

**Figure 6:**
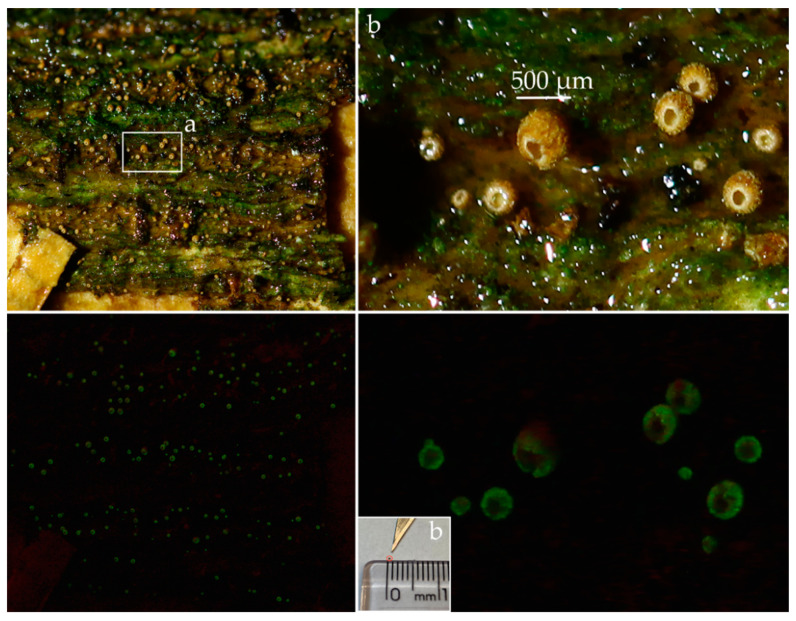
*Eoscyphella luciurceolata* basidiomata in light (above) and dark (below) on removed bark of “fumeiro” tree (*Solanum swartzianum*). Note that mushrooms are in wetter conditions and all of them emit light. (**a**) A dried mushroom is shown next to a scalpel blade to demonstrate its size; (**b**) FBIPBio 93.20220802 (Paratype–FIFUNGI00249). Photos by Adão Henrique Rosa Domingos and Isaias Santos.

**Figure 7:**
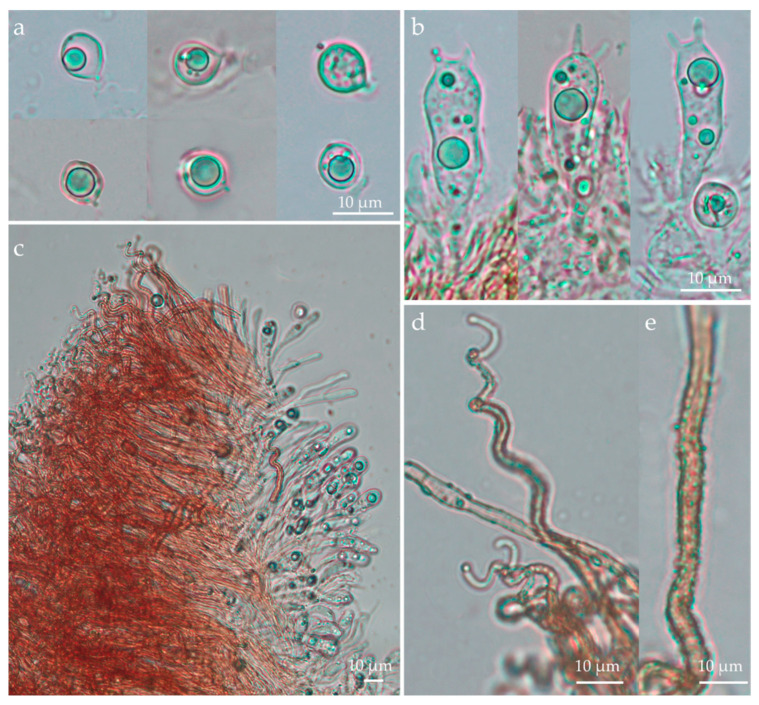
*Eoscyphella luciurceolata* (FBIPBio 96.20230322, holotype–FIFUNGI0001). (**a**) Basidiospores; (**b**) basidia; (**c**) hymenium and external surface; (**d**,**e**) external hyphae. Photos by Alexandre G. S. Silva-Filho and Cristiano C. Nascimento.

**Figure 8:**
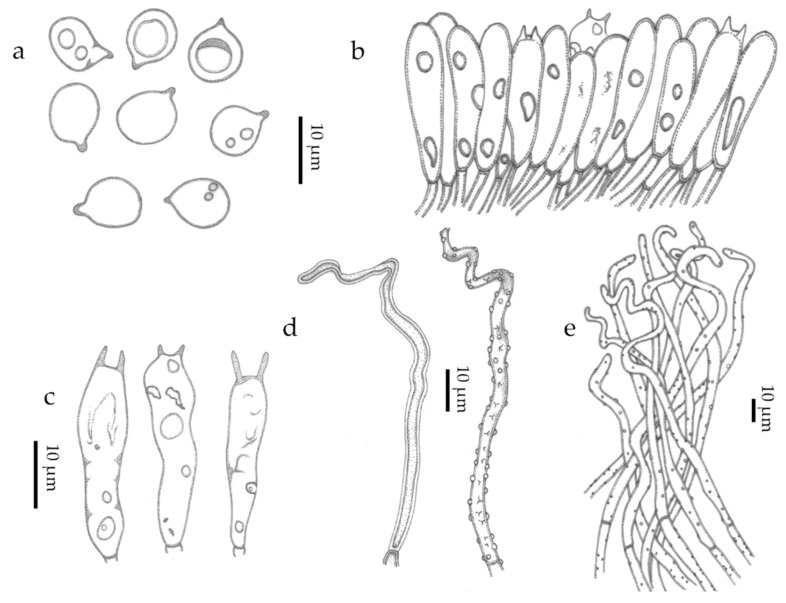
*Eoscyphella luciurceolata* (FBIPBio 96.20230322, holotype-FIFUNGI0001. (**a**) Basidiospores; (**b**) hymenium with basidia and basidioles; (**c**) basidia; (**d**,**e**) external hyphae. Drawings: original by Alexandre G. S. Silva-Filho and inked by K. Sousa.

***Eoscyphella luciurceolata*** Silva-Filho, Stevani & Desjardin sp. nov. Figure 5, Figure 6, Figure 7 and Figure 8.

MycoBank: MB 849404 

Etymology: *Luci* = light (Latin); *urceolus* = diminutive of urceus “pitcher” (Latin), in reference to urceolate shape of the receptacle. Since bioluminescent and non-bioluminescent species occur in the genus, the prefix “Luci” is here applied to differentiate this new species from putative non-bioluminescent ones.

Holotype: BRAZIL, São Paulo state, Eldorado, approximately 500 m west of the entrance to the “Caverna do Diabo” (Devil’s Cave) State Park, but still in the buffered conservation area, on a single “fumeiro” tree (*Solanum swartzianum* Roem. & Schult.), 24°38′14.0100″ S and 48°24′37.6812″ W, alt. 546 m, 22 March 2023, FBIPBio 96.20230322, leg. Isaias Santos, Adão Henrique Rosa Domingos, Olavo H. P. Della-Torre (FIFUNGI0001, holotype!) GenBank [ITS rDNA]: OR230671, [LSU rDNA]: OR230673.

Diagnosis: *Eoscyphella luciurceolata* differs from other known species of cyphelloid fungi by the following combination of characters: receptacle vasiform to urceolate without conspicuous long hairs; external hyphae cylindrical, sinuous, coiled to conspicuously spiraled, pigmented, weakly to densely incrusted overall, less so near their tips, with small globular crystals; basidiospores subglobose to ovoid or broadly ellipsoid; basidia cylindrical to subclavate, 2-spored (rarely 4-spored); hymenial cystidia absent; clamp connections absent.

Basidiomata scattered (Figure 5 and Figure 6). Receptacle 0.3–0.5 mm tall, 0.2–0.3 mm diam, vasiform to urceolate, sessile (astipitate), with distinct opening; external surface dull, dry, felted to appressed-pubescent, conspicuous long hairs absent, pale yellow (2A3) to greyish yellow (2B3, 4B5) or greyish orange (5B4), white (1A–B1) near the distal opening (Figure 5c and Figure 6b; subiculum absent; hymenial surface greyish yellow (2C4), smooth. External hyphae 60–128 × 2.0–4.0 μm, cylindrical, sinuous to coiled, yellowish brown to brownish orange in water or KOH, weakly to densely incrusted overall, less so near the tips, with small globular crystals, thick-walled (0.5–1.5 um thick), thinner near the tip, inamyloid, non-gelatinous, unclamped; terminal cells narrowed towards the tip to 1.5–2.0 µm diam, tips hyaline to pale yellowish brown, obtuse to subacute, those at the margin of the pore hyaline and conspicuously spiraled (Figure 7c–e and Figure 8d–e); dendrohyphidia absent. Trama composed of an interwoven layer of irregularly cylindrical to inflated, short-celled hyphae 3.0–9.5 µm diam, hyaline to pale yellowish brown, much-branched, non-incrusted, non-gelatinous, thin- to thick-walled (0–0.5 µm thick), unclamped (Figure 7c). Subhymenial layer composed of cylindrical hyphae 3.0–4.0 μm diam, hyaline, thin- to thick-walled (0.5–1.5 µm thick), unclamped. Basidiospores (6.5–)7.5–9.5 × (5.5–)6.5–8(–9.5) μm [xm = 8.56 ± 0.13 × 7.35 ± 0.29 µm, xr = 8.5–8.7 × 7.1–7.6 µm, Q = 1.0–1.6, Qm = 1.18 ± 0.05, n = 60, s = 3], subglobose to ovoid or broadly ellipsoid, predominantly subglobose, smooth, hyaline, inamyloid, sometimes one- or two-guttulate, hilar appendix up to 1 μm long, thin- or thick-walled (0.5–1.0 µm) at maturity (Figure 7a and Figure 8a). Basidia 22–32 × 7.0–10.0 μm, cylindrical to subclavate, two-spored, rarely four-spored, hyaline, sometimes with refringent contents, unclamped; sterigmata up to 12 μm long (Figure 7b and Figure 8b). Basidioles subclavate (Figure 7c and Figure 8b). Hymenial cystidia absent (Figure 7c). Clamp connections absent in all tissues examined. Bioluminescence: emitting yellowish green light only in a narrow band around the pore margin of the receptacle; water droplets likely magnify the light (Figure 5 and Figure 6).

Additional specimens examined: BRAZIL, São Paulo state, Eldorado, exact same location, and tree described above, 2 August 2023, FBIPBio 93.20220802, leg. Isaias Santos, Adão Henrique Rosa Domingos, Olavo H. P. Della-Torre (FIFUNGI00249, Paratype!); *ibid*, 20 September 2022, FBIPBio 94.20220920 (FIFUNGI00250, Paratype!) GenBank [ITS rDNA]: OR230672, [LSU rDNA] OR230674.

Habitat and known distribution: On bark of “fumeiro” tree (*Solanum swartzianum*) in the Atlantic Rainforest, southern Brazil. Known only from the type locality.

Notes: When morphologically compared with other cyphelloid species, *Maireina spiralis* (Coker) W.B. Cooke has external hyphae with spiral tips, differing from *E. luciurceolata* in the clamped hyphae and with longer (11–15 µm long) ellipsoid basidiospores [43]. *Maireina afibulata* Bodensteiner and *Ma. pseudochracea* W.B. Cooke do not produce clamp connection, but the first has smaller basidiospores (5–6(–6.5) × 3–4 μm) and both have straight external hyphae and produce smaller basidia (6–23 × 5–6.5 µm in *Ma. afibulata*; 17.5 × 5.8 µm in *Ma. pseudochracea*) with four sterigma [43,53].

## 4. Discussion

The morphological delimitation of *Merismodes*, *Cyphellopsis*, and *Maireina* has been the cause of debates about the morphological limits of these genera [43,51,57,62,80,81,82,83]. Reid [81] considered *Cyphellopsis* and *Maireina* as synonyms and suggested that the depth of the cavity that lined the hymenium is a character insufficient for the separation of *Cyphellopsis* (=*Maireina*) and *Merismodes.* Singer [83] synonymized the genus *Cyphellopsis* and *Maireina* with *Merismodes* and listed both *Maireina* and *Cyphellopsis* as sections. The first broad research on cyphelloid fungi based on molecular phylogenetic analyses resolved *Merismodes* and *Cyphellopsis* as a monophyletic group, recognizing them as a single genus [45]. Another broad study of *Maireina* without molecular data led Bodensterner [53,57] to recognize the genus *Maireina* as an independent lineage from *Merismodes* and *Cyphellopsis*. Knudsen and Vesterholt [61] recognized *Cyphellopsis*, *Maireina*, and *Phaeocyphellopsis* as synonyms of *Merismodes,* providing a broad description for the genus. The first works to describe new species of *Maireina* based in-part on molecular data are those of Læssøe et al. [54] and Mombert [55]. In both, the sequences of *Maireina* clustered with *Merismodes* and *Cyphellopsis* in a large clade, making it possible to determine the phylogenetic position within Cyphellopsidaceae. Our phylogenetic analyses in separate and combined LSU rDNA and ITS rDNA recognized *Merismodes*, *Cyphellopsis*, and *Maireina* as a monophyletic group, supporting the proposal of Knudsen and Vesterholt [61] for a broad morphological concept of *Merismodes*. The samples and sequences of *Me. monacha*, type species of *Maireina*, first studied by Mombert [55] were extremely important for the recognition and the phylogenetic positioning of the genus *Maireina*. Although the sequences are not of the holotype specimen, the collection is from a region very close to the type locality, and the morphological description agrees with the complete redescription presented by Cooke [43].

Our cyphelloid bioluminescent samples were initially identified within the morphological concept of *Maireina sensu* Bodensteiner [53,57]. However, our phylograms (Figure 1a, Figure 2 and Figure 3) showed a phylogenetic distance between *E. luciurceolata* and *Me. monacha,* which are only 90.6% to 90.7% similar in the LSU rDNA and 64.6% to 65.9% similar in the ITS rDNA. *Eoscyphella* is closely related to the genus *Woldmaria* in our analyses, but the included taxa are 7.3% to 7.4% divergent in their LSU rDNA sequences, a high value considering a similarity threshold of around 96.91% to discriminate genera using LSU rDNA in Basidiomycota [84]. These data and results support the proposition of a new cyphelloid genus and distinct molecular lineage. Additionally, *Eoscyphella* is also morphologically well delimited with receptacles that lack conspicuous long hairs, subglobose to broadly ellipsoid basidiospores, frequently bi-spored basidia, unclamped hyphae, and weakly to densely incrusted overall external hyphae, which are always pigmented and encrusted at the tips.

Regarding the cyphelloid genera within Agaricomycetes, our LSU rDNA analyses retrieved 11 lineages of cyphelloid fungi and the phylogenetic relationship of the cyphelloid genera agrees with recent phylogenetic studies [45,50,56]. However, we highlight that sequences of the collection PB327 named as *Calathella columbiana* appear in different positions and for this reason were excluded from the combined analyses: in the ITS rDNA tree within Cyphellopsidaceae (Figure 2), and in the LSU rDNA tree (Figure 1c) in a clade close to representatives of Entolomataceae. Additionally, *Phaeosolenia densa* (Berk.) W.B. Cooke was shown by Bodensteiner et al. [45] to be close to the genus *Tubaria* (W.G. Sm.) Gillet, whilst in our analyses, it forms an isolated clade without support (Figure 1c).

Desjardin et al. [4] performed the second review of bioluminescent fungi worldwide, referring 64 luminescent species into three lineages, *Armillaria,* Mycenoid, and *Omphalotus*, indicating that *Gerronema viridilucens* and *Mycena lucentipes* do not belong to the Mycenoid lineage. Later, Desjardin et al. [24] referred *G. viridilucens* and *M. lucentipes* to a new and unnamed lineage, which was later named the Lucentipes lineage by Oliveira et al. [35]. Our LSU rDNA phylogram (Figure 1b) shows and confirms *G. viridilucens* plus *M. lucentipes* as a separate bioluminescent lineage. The *Eoscyphella* lineage is here recognized as a new and fifth bioluminescent lineage in Cyphellopsidaceae (Figure 1a).

From previous phylogenetic analyses, *G. viridilucens* has been proposed within Porotheleaceae [70,71]. However, our LSU rDNA phylogram (Figure 1b) showed Porotheleaceae, represented by type species of the genus *Hydropus* [*Hydropus fuliginarius* (Batsch) Singer, AF261368], forming a well-supported clade (94%, BS, 0.99 BPP) that harbors most of the species of *Gerronema* Singer, except *G. viridilucens*, which clustered with sequences of *M. lucentipes*, *Atheniella rutilla* (NG153951), *Hydropus trichoderma* (MK278154), *Mycena* cf. *quiniaultensis* (EU681183), and *Mycopan scabripes* (MK278154) in a clade phylogenetically distant from Porotheleaceae. Vizzini et al. [70] showed the genera *Acanthocorticium* Baltazar, Gorjón & Rajchenb.; *Athelia* Pers.; *Atheniella* Redhead, Moncalvo, Vilgalys, Desjardin & B.A. Perry; *Baeospora* Singer; *Calyptella*; *Campanophyllum* Cifuentes & R.H. Petersen; *Cheimonophyllum* Singer; *Chondrostereum* Pouzar; *Cyphella*; *Granulobasidium* Jülich; *Gloeostereum* S. Ito & S. Imai; *Mycopan* Redhead, Moncalvo & Vilgalys; *Pleurella* E. Horak; *Henningsomyces*; and *Rectipilus* as part of the *Henningsomyces/Rectipius/Acanthocorticium* clade, with all accommodated in Cyphellaceae. Due to the close relationship between *Atheniella* and *Mycopan*, most of the genera of Cyphellaceae sensu Vizzini et al. [70] were included in our phylogeny in order to confirm the phylogenetic position of *G. viridilucens* plus *Mycena lucentipes*. However, in our LSU rDNA analyses (Figure 1a,b), Cyphellaceae sensu Vizzini et al. [70] was retrieved as a polyphyletic group, with representatives grouped into five different clades. In the LSU rDNA phylogram (Figure 1b) Cyphellaceae can be well represented by the clade with sequences of *Cyphella digitalis* (Alb. & Schwein.) Fr. (AY29293175 and AY635771), *Cheimonophyllum candidissimum* (Sacc.) Singer (DQ457654), and *Campanophyllum proboscideum* (Fr.) Cifuentes & R.H. Petersen (AY230866). Thus, it is confirmed that *G. viridilucens* and *Mycena lucentipes* are positioned neither in *Porothelleaceae* nor in *Cyphellaceae*.

## 5. Conclusions

Our systematic study confirms the findings of previous studies regarding the existence of multiple bioluminescent lineages in Agaricales. All bioluminescent fungi have currently been described in suborder Marasmiineae Aime, Dentinger & Gaya. The newly described *Eoscyphella luciurceolata* was confirmed from molecular phylogenies in the family Cyphellopsidaceae, currently accepted within the suborder Schizophyllineaeae Aime, Dentinger & Gaya [62]. Additionally, our study reveals a new lineage within a group primarily consisting of reduced forms. Fungal bioluminescence engages in a cyclical process of biosynthesis known as the Caffeic Acid Cycle (CAC), which relies on a sequence of four consecutive enzymes: hispidin synthase (HispS), hispidin-3-hydroxylase (H3H), luciferase (Luz), and caffeylpyruvate hydrolase (CPH) [3]. At present, there are limited genomic data concerning bioluminescent fungi in the existing literature [85], with the majority originating from the Mycenoid and *Armillaria* lineages. By identifying this recently discovered bioluminescent lineage and uncovering the sequences of the *hisps*, *h3h*, *luz*, and *cph* genes, there is potential for enhancing our understanding of the evolutionary progression of the bioluminescent trait within the fungal kingdom.

A high diversity of bioluminescent fungi has been discovered in Brazil, with 23 species (including our new described species) reported theretofore, see [86]. In the Brazilian Atlantic Rainforest, new species of bioluminescent fungi have been described or reported, e.g., [87], with emphasis to the southwestern portion of the state of São Paulo, the same area where *E. luciurceolata* was found and where another 12 species of Mycenoid and Lucentipes lineage taxa have already been described or reported [22,23,24,25]. Even so, new bioluminescent samples collected at the same area are currently in the process of molecular and morphological characterization and may represent taxonomic novelties, demonstrating that the Atlantic Rainforest in the southwestern region of the São Paulo state is one of the most studied areas of bioluminescent fungi and may represent a biodiversity hot spot for these organisms.

## Figures and Tables

**Figure 4:**
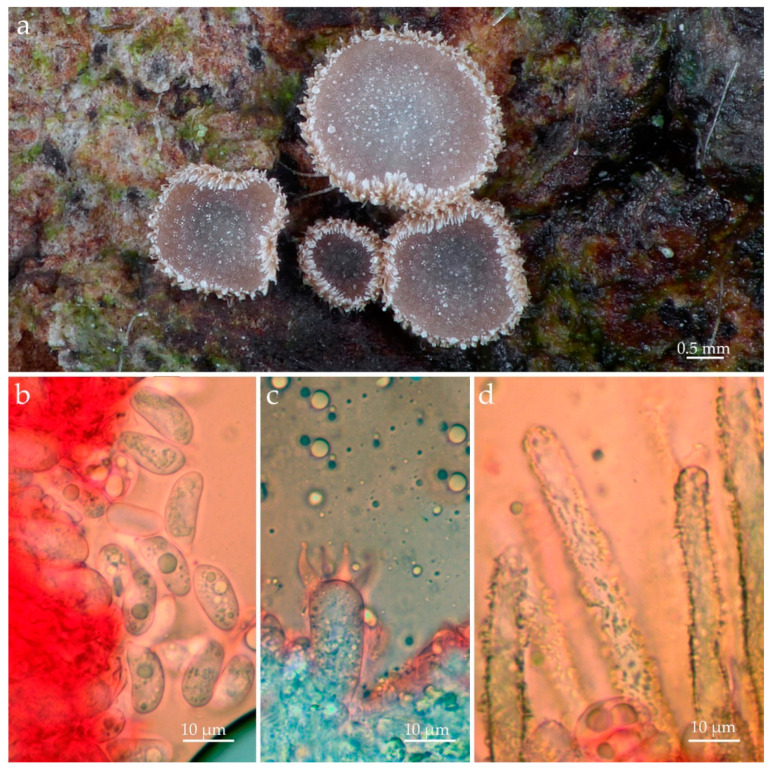
*Merismodes monacha* (ALV30536, Epitype–PC0142589). (**a**) Basidiomata in situ; (**b**) basidiospores; (**c**) basidium; (**d**) external hyphae. Photos by Andgelo Mombert.

## Data Availability

The DNA sequence data obtained in this study were deposited at GenBank. The accession numbers can be found in the trees and in Supplementary Table S1. This study is according to the Brazilian legislation on access to biodiversity and is registered in the “Sistema Nacional de Gestão do Patrimônio Genético e do Conhecimento Tradicional Associado” (SisGen #A5A80A7).

## Supplementary Materials

The following supporting information can be downloaded at: https://www.mdpi.com/2309-608X/9/10/1004/s1, Table S1: List of specimens; culture, herbarium access number, isolate, strain, or voucher collection (V); and GenBank accession numbers.
