# Correction: Silva-Filho et al. *Eoscyphella luciurceolata* gen. and sp. nov. (Agaricomycetes) Shed Light on Cyphellopsidaceae with a New Lineage of Bioluminescent Fungi. *J. Fungi* 2023, *9*, 1004

**DOI:** 10.3390/jof9121189

**Published:** 2023-12-13

**Authors:** Alexandre G. S. Silva-Filho, Andgelo Mombert, Cristiano C. Nascimento, Bianca B. Nóbrega, Douglas M. M. Soares, Ana G. S. Martins, Adão H. R. Domingos, Isaias Santos, Olavo H. P. Della-Torre, Brian A. Perry, Dennis E. Desjardin, Cassius V. Stevani, Nelson Menolli

**Affiliations:** 1 IFungiLab, Departamento de Ciências da Natureza e Matemática (DCM), Subárea de Biologia (SAB), Instituto Federal de Educação, Ciência e Tecnologia de São Paulo (IFSP), Campus São Paulo (SPO), São Paulo 01109-010, SP, Brazil; silvafilhoags@gmail.com (A.G.S.S.-F.); cristiano.nascimento@ifpi.edu.br (C.C.N.); 2 Independent Researcher, 25640 Corcelle-Mieslot, France; mombertan@gmail.com; 3 Departamento de Bioquímica, Instituto de Química, Universidade de São Paulo, São Paulo 05508-000, SP, Brazil; bianca.barros.nobrega@usp.br; 4 Departamento de Química Fundamental, Instituto de Química, Universidade de São Paulo, São Paulo 05508-000, SP, Brazil; douglas@iq.usp.br; 5 Instituto de Pesquisa da Biodiversidade (IPBio), Iporanga 18330-000, SP, Brazil; anaglaucia@ipbio.org.br (A.G.S.M.); henrique.domingos@ipbio.org.br (A.H.R.D.); tatubio@hotmail.com (I.S.); olavopetrucci@gmail.com (O.H.P.D.-T.); 6 Department of Biological Sciences, California State University, East Bay, Hayward, CA 94542, USA; brian.perry@csueastbay.edu; 7 Department of Biology, San Francisco State University, San Francisco, CA 94132, USA; ded@sfsu.edu

## Error in Figure

In the original publication [1], there was a mistake in Figure 1d as published. The sequences KY418873 and KF530568 from *Cyptotrama asprata* (Berk.) Redhead & Ginns and *Xerula strigosa* Zhu L. Yang, L. Wang & G.M. Muell. respectively were erroneously noted by symbol ⚫︎. The corrected Figure 1d appears below.


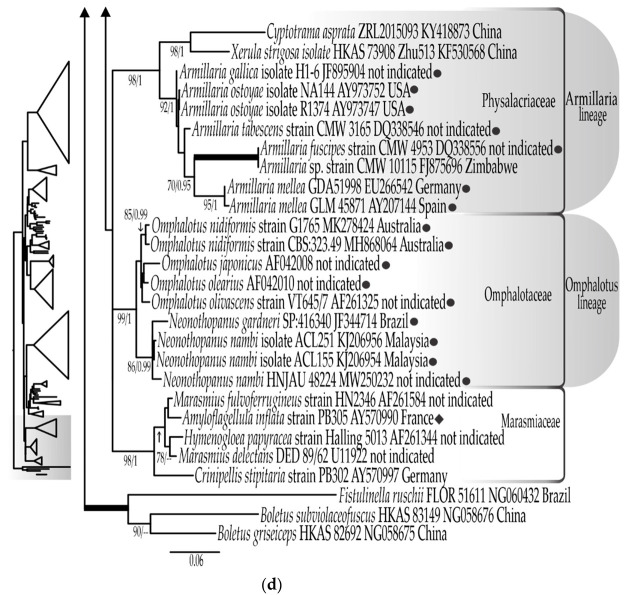


## Text Correction

There was an error in the original publication. We did not include acknowledgment to Carlos Roberto Silva Moraes. A correction has been made to Acknowledgments. 

**Acknowledgments:** The authors thank Alexandro Andrade and Jefferson Góis for the initial taxonomic support, Suzana Ehlin Martins for the taxonomic identification of the host plant, Dimitrios Floudas for suggesting the Greek prefix of the new generic name and also for helping to compose the etymology of the new genus, Carlos Roberto Silva Moraes (also known as Duco) for granting us access to the collection site, and the anonymous reviewers for improvements to the original manuscript. We are also grateful to “Fundação Florestal” and “Secretaria do Meio Ambiente do Estado de São Paulo” for the collection licenses (process #260108-010.245/2017).

The authors apologize for any inconvenience caused and state that the scientific conclusions are unaffected. The original publication has also been updated.
