# Supplementary material for: Eoscyphella luciurceolata gen. and sp. nov. (Agaricomycetes) Shed Light on Cyphellopsidaceae with a New Lineage of Bioluminescent Fungi
Source: J Fungi (Basel). 2023 Oct 12;9(10):1004. doi: 10.3390/jof9101004 (PMC10608165; doi:10.3390/jof9101004)
Supplement: Supplementary file 1 [file jof-09-01004-s001.zip › jof-2537218-supplementary.pdf]

Supplementary materials

Supplementary Table S1: List of specimens; culture, herbarium access number, isolate, strain or voucher collection (V); and GenBank accession numbers.

Species	Clone (Cl), culture (C), herbarium access number (H), isolate (I), strain (S) or voucher collection (V)	LSU rDNA accession nº	ITS rDNA accession nº
Acanthocorticium brueggemannii	(V) JMB2122	KT275195	-
Acanthocorticium brueggemannii	(V) JMB2621	KT275196	-
Akenomyces costatus	(S) CBS:371.86	MH873657	-
Akenomyces costatus	(S) CBS:513.83	MH873350	MH861635
Amyloflagellula inflata	(S) PB305	AY570990	-
Armillaria fuscipes	(S) CMW 4953	DQ338556	-
Armillaria gallica	(I) H1-6	JF895904	-
Armillaria mellea	(V) GLM 45871	AY207144	-
Armillaria mellea	(V) GDA51998	EU266542	-
Armillaria ostoyae	(V) R1374	AY973747	-
Armillaria ostoyae	(V) NA144	AY973752	-
Armillaria sp.	(S) CM 10115	FJ875696	-
Armillaria tabescens	(S) CMW 3165	DQ338546	-
Athelia bombacina	(S) ATCC 20629	AF279377	-
Atheniella rutila	(V) FFAAS 0354	NG 153951	-
Baeospora myosura	(I) AFTOL-ID 1799	DQ457648	-
Boletus griseiceps	(V) HKAS 82692	NG 058675	-
Boletus subviolaceofuscus	(V) HKAS 83149	NG 058676	-
Bulbillomyces farinosus	(V) FO24378	AJ406578	-
Calathella columbiana	(S) PB327	AY570993	AY571028
Calathella gayana	(S) ZT8836	AY572005	-
Calathella mangrovei	(S) 1-31-0	-	AY571029
Calyptella capula	(S) CBS:485.86	AY570994	-
Calyptella capula	(S) PB315	AY570995	-
Calyptella sp.	(V) 7110217	AB512346	-
Campanophyllum proboscideum	(V) TENN56402	AY230866	-
Campanophyllum proboscideum	(V) TENN56427	AY230867	-
Cheimonophyllum candidissimum	(I) AFTOL-ID 1765	DQ457654	-
Chondrostereum purpureum	(V) C277	KR264909	-
Chondrostereum purpureum	(V) GEL5348	AJ406563	-
Chondrostereum purpureum	(I) CBS 350 53	MH868775	-
Chromocyphella lamellata	(V) AH 45802	MF623831	-
Clitocybula oculus	(S) DAOM195995	AF261367	-
Crepidotus subfulviceps	(I) MCA 3276	FJ947117	-

Crinipellis stipitaria	(S) PB302	AY570997	-
Cunninghammyces sp.	(V) He5311	-	MW557940
Cunninghammyces sp.	(V) He5313	-	MW557941
Cyphella digitalis	(I) Thorn-617	AY293175	-
Cyphella digitalis	(I) AFTOL-ID 663	AY635771	-
Cyphelloporia bialoviensis	(V) KRAM F-59691	ON391555	-
Cyphelloporia bialoviensis	(S) G1780	MK278141	-
Cyptotrama asprata	(V) ZRL2015093	KY418873	-
Dendrothele acerina	(S) G0632	MK277925	-
Dendrothele alliacea	(S) G0488	MK277926	-
Dendrothele bispora	(S) CBS:962.96	-	OM238172
Dendrothele griseocana	(C) CBS:340.66	-	MH858816
Dendrothele incrustans	(S) HHB-19092	-	MW740330
Dendrothele microspora	(S) FP.101998	-	OQ694474
Dendrothele minutissima	(S) G1841	MK277929	-
Dendrothele nivosa	(V BHI-F632	-	MF161308
Dendrothele nivosa	(V) iNat62633580	-	ON129313
Dendrothele sp.	(S) G1907	MK277935	-
Dendrothele sp.	(S) G1876	MK277934	-
Entoloma bloxamii	(S) TB6117	AF261289	-
Entoloma eulividum	(S) TB6807	AF261295	-
Eoscyphella luciurceolata	**(V) FBIPBio94**	**OR230674**	**OR230672**
Eoscyphella luciurceolata	**(V) FBIPBio96**	**OR230673**	**OR230671**
***Eoscyphella* sp.**	**(V) FPIBio01**	-	**OR260255**
Filoboletus manipularis	(I) ACL252	KJ206957	-
Filoboletus manipularis	(I) KUBOT-KRMK-2020-53	MW444998	-
Filoboletus pallescens	(V) BAP 654	MH385337	-
Filoboletus pallescens	(V) DED 8303	MH385336	-
Fistulinella ruschii	(H) FLOR 51611	NG_060432	-
Flagelloscypha austrofilicis	(V) PDD 105591	KM975368	KM975436
Flagelloscypha eruciformis	(V) K(M) 181434	-	MZ159475
Flagelloscypha eruciformis	(V) MH-2018-0187	-	MK434305
Flagelloscypha eruciformis	(V) MH-2018-0190	-	MK434306
Flagelloscypha japonica	(V) NBRC 101830	AB455964	LC146734
Flagelloscypha minutissima	(S) CBS:823.88	AY571006	-
Flagelloscypha minutissima	(C) M01452:264	-	LR872900
Flagelloscypha sp.	(V) Horak 9544	AY571007	AY571041
Flagelloscypha sp.	(V) FLAS-F-70981	OP932039	OP932048
Flagelloscypha sp.	(V) NBRC 104516	AB455962	-
Flagelloscypha sp.	(V) P. del j.	JX243756	-
Flammula alnicola	(I) AFTOL-ID 1501	DQ457666	-
Galerina sp.	(V) PR6574	HQ827183	-

Gerronema baishanzuense	(V) FFAAS0359	OL985984	-
Gerronema indigoticum	(V) HMJAU47636	MK693732	-
Gerronema keralense	(V) CAL 1666	NG_064531	-
Gerronema kuruvense	(V) CAL 1665	NG_064530	-
Gerronema nemorale	(V) FFAAS0379	OL985998	-
Gerronema nemorale	(V) FFAAS0377	OL985997	-
Gerronema sp.	(V) OKM27143	AF261365	-
Gerronema strombodes	(S) JEJ 580	AF261364	-
Gerronema subclavatum	(I) Redhead 5175	U66434	-
Gerronema viridilucens	(V) DED7822	OR449361	-
Gerronema wildpretii	(V) BRNM 788347	LT854043	-
Gerronema xanthophyllum	(V) PRM 924657	LT854023	-
Gerronema zhujian	(V) FFAAS0364	OL985994	-
Gerronema zhujian	(V) FFAAS037	OL985995	-
Gloeostereum sp.	(S) CBS 145006	MN266884	-
Granulobasidium vellereum	(I) AFTOL-ID 887	AY745729	-
Halocyphina villosa	(V) NBRC 32086	AB455965	-
Halocyphina villosa	(S) IFO32088	-	AY571042
Hebeloma microspora	(V) E Rebaudengo 5-V-1976	MF039240	-
Henningsomyces candidus	(S) PB338	AY571008	-
Henningsomyces candidus	(S) R.G.Thorn156	AF287864	-
Henningsomyces puber	(S) GUA-307	AY571009	-
Henningsomyces sp.	(V) FP-105017	AY571010	-
Hydropus fuliginarius	(V) DAOM196062	AF261368	-
Hydropus trichoderma	(S) G0347	MK278158	-
Hymenogloea papyracea	(S) Halling 5013	AF261344	-
Lachnella alboviolascens	(S) PB332	AY571012	-
Lachnella alboviolascens	(S) DAOM223321	AF261475	-
Lachnella alboviolascens	(S) MO315410	-	MH558281
Lachnella sp.	(I) ES104	-	MN218811
Lachnella tiliae	(S) G0887	MK277903	-
Lachnella villosa	(S) CBS:609.87	DQ097347	-
Lachnella villosa	(S) PB322	AY571014	AY571050
Lachnella villosa	(S) PB321	AY571013	AY571049
Lachnella villosa	(V) CCJ 1547	DQ071724	-
Macrotyphula fistulosa	(V) TUB 011469	DQ071735	-
Maireina filipendula	(V) TL14226	KX772745	KX772745
Maireina filipendula	(V) TL2015-724890	KX772746	KX772746
Maireina monacha	(V) ALV30536	OR197581	OP099548
Maireina subsphaerosphora	(V) ALV34117	OR197528	-
Maireina subsphaerosphora	(V ALV27634	OP099548	OP099549
Marasmius delectans	(I) DED 89/62	U11922	-

Marasmius fulvoferrugineus	(S) HN2346	AF261584	-
Merismodes anomala as 'Cyphellopsis'	(V) TL2015-719433	KX772747	KX772748
Merismodes anomala as 'Cyphellopsis'	(S) PB323	AY570999	AY571036
Merismodes anomala as 'Cyphellopsis'	(S) PB318	AY570998	AY571036
Merismodes anomala as 'Cyphellopsis'	(S) PB333	AY571000	-
Merismodes anomala as 'Cyphellopsis'	(S) CBS:151.79	AF426955	AY571034
Merismodes anomala as 'Cyphellopsis'	(V) GEL4169	AJ406522	-
Merismodes fasciculata	(S) PB342	AY571016	AY571052
Merismodes fasciculata	(S) G1064	MK278379	-
Merismodes fasciculata	(S) HHB-11894	AY571051	-
Merismodes fasciculata	(V) K(M)194471	MZ159522	-
Merismodes fasciculata	(I) HHB-11894	AY571015	-
Merismodes sp.	(V) Alden Dirks ACD0273	MZ919217	-
Mycena caeruleomarginata	(V) FFAAS 0357	NG 153993	-
Mycena cahaya	(I) ACL134	KJ206950	-
Mycena cf. *Quiniaultensis*	(V) OSC 67121	EU681183	-
Mycena chlorophos	(I) ACL 259	JX975221	-
Mycena chlorophos	(I) ACL 055	JX975220	-
Mycena crocata	(V) GLM 45968	AY207241	-
Mycena galericulata	(V) GLM 45970	AY207251	-
Mycena galopus	(V) GLM 45971	AY207250	-
Mycena haematopus	(V) GLM 45972	AY207252	-
Mycena illuminans	(I) ACL 175	JX975218	-
Mycena illuminans	(I) ACL 161	JX975217	-
Mycena indigotica	(S) WEI16-475	MH063432	-
Mycena lucentipes	(V) DED7828	OR343215	-
Mycena maculata	(V) GLM 45974	AY207254	-
Mycena noctilucens	(I) ACL054	KJ206947	-
Mycena olivaceomarginata	(V) GLM 45976	AY207255	-
Mycena polygramma	(V) TUB 011575	DQ071707	-
Mycena pura	(I) TM02 201	EU522743	-
Mycena sanguinolenta	(V) GLM 45982	AY207257	-
Mycena seminau	(I) ACL136	KJ206952	-
Mycena sinar	(I) ACL092	KJ206948	-
Mycena sp.	(V) JMCR 32	AF261362	-
Mycena sp.	(I) ACL133	KJ206949	-
Mycena tintinnabulum	(V) GLM 45983	AY207258	-
Mycena zephirus	(V) GLM 45984	AY207259	-
Mycopan scabripes	(S) G0261 MK278154	MK278154	-
Mycopan scabripes	(I) DAOM192847	AF042635	-
Neonothopanus gardneri	(H) SP:416340	JF344714	-
Neonothopanus nambi	(I) ACL251	KJ206956	-

Neonothopanus nambi	(I) ACL155	KJ206954	-
Neonothopanus nambi	(V) HNJAU 48224	MW250232	-
Nia sp.	(V) MF 1330	MK491335	
Nia sp.	(S) CBS H-23851	MK491336	OQ555325
Nia vibrissa	(V) NBRC 32090	AB455968	-
Nia vibrissa	(I) FCUL170907CF6	MG597153	-
Nia vibrissa	(V) FCUL070108CF9	MG597151	-
Nia vibrissa	(V) NBRC 32089	AB455967	-
Nia vibrissa	(I) CBS:1198.15	-	OQ694464
Nia vibrissa	(I) REG M200	-	AY57105
Omphalotus japonicus	(I) 456	AF042008	-
Omphalotus nidiformis	(S) CBS:323.49	MH868064	-
Omphalotus nidiformis	(S) G1765	MK278424	-
Omphalotus olearius	(I) CBS:141.34	AF042010	-
Omphalotus olivascens	(S) VT645 7	AF261325	-
Panaeolina foenisecii	(I) J152	AF041537	-
Panellus crassiporus	(V) Dai 23664	ON074730	-
Panellus crassiporus	(V) BJFC 031637	NG_088354	-
Panellus luminescens	(I) ACL205	KJ206955	-
Panellus pusillus	(S) RV PR98 36	AF261425	-
Panellus stipticus strain	(S) CBS 389 50	MH868192	-
Panellus stypticus	(S) GLM 51801	AY398745	-
Pellidiscus pallidus	(S) C58178	AY571017	-
Pellidiscus sp.	(Cl) H06M7K43	HF676376	-
Peyronelina glomerulata	(V) NBRC 104517	AB455955	-
Peyronelina glomerulata	(V) NBRC 104522	AB455961	-
Peyronelina glomerulata	(V) NBRC 104521	AB455959	-
Peyronelina glomerulata	(V) NBRC 102381	AB455958	-
Phaeosolenia densa	(S) C61963	AY571019	-
Phaeosolenia densa	(S) C61839	AY571018	-
Phyllotopsis nidulans	(V) TENN F-69019	KP026224	-
Phyllotopsis nidulans	(I) TFB11482	KP026220	-
Pleurocybella porrigens	(I) AFTOL-ID 2001	EF537894	-
Pleurocybella porrigens	(V) TUB 012154	DQ071737	-
Pleurotus eryngii	(I) d9	AY524788	-
Pleurotus nebrodensis	(S) ACCC 51060	EU365659	-
Pleurotus nebrodensis	(I) P151	MG282550	-
Pleurotus ostreatus	(S) CBS 375 51	EU365665	-
Pleurotus ostreatus	(V) TUB 011571	DQ071722	-
Pleurotus pulmonarius	(I) d3	AY524787	-
Pleurotus pulmonarius	(V) BORHF0429	MH178092	-
Pouzarella nodospora	(S) TB5716	AF261308	-

Pseudolasiobolus minutissimus	(V) G M 2017-11-19	MN630583	MN630583
Rectipilus fasciculatus	(V) GEL4482	AJ406553	-
Rectipilus idahoensis	(S) PB313	AY571020	-
Rectipilus natalensis	(S) PB312	AY571021	-
Rectipilus sp.	(V) G1859	MK277907	-
Rectipilus sp.	(V) G1863	MK277909	-
Resupinatus alboniger	(S) G0079	MK278439	-
Resupinatus conspersus	(V) C61852	AY571024	-
Rhodocybe mundula	(S) TB4698	AF261284	-
Roridomyces roridus	(V) NOFQB24 Q35	LC757539	-
Roridomyces roridus	(V) GLM 45980	AY207298	-
Roridomyces roridus	(S) DAOM215019	AF261408	-
Simocybe rhabarbarina	(V) LUG 19069	NG 059999	-
Stigmatolemma urceolatum	(S) G0373	MK278578	-
Stromatoscypha fimbriatum	(S) HC.10/11/98.C	AF261370	-
Stromatoscypha fimbriatum	(S) FP102067	AF261371	-
Trogia infundibuliformis	(S) KUN HKAS63661	JQ031780	-
Trogia infundibuliformis	(S) KUN HKAS56709	JQ031781	-
Trogia venenata	(S) KUN HKAS54710	JQ031778	-
Trogia venenata	(S) KUN HKAS56679	JQ031779	-
Tubaria hiemalis	(V) MCA385	AF205689	-
Tubaria keralensis	(V) CAL 1311	KX585250	-
Tubaria peculiaris	(V) PDD 98277	KY827260	-
Tubaria peculiaris	(V) PDD 72769	KY827259	-
Woldmaria crocea	(S) G1864 MK278664	MK278664	-
Woldmaria crocea	(S) NH 10.23.95	AY571026	-
Xerula strigosa	(V) Zhu513	KF530568	-
